# Sales of Electronic Nicotine Delivery Systems (ENDS) and Cigarette Sales in the USA: A Trend Break Analysis

**DOI:** 10.1007/s10603-022-09533-4

**Published:** 2023-01-16

**Authors:** A. Selya, R. Wissmann, S. Shiffman, S. Chandra, M. Sembower, J. Joselow, S. Kim

**Affiliations:** 1 PinneyAssociates, Inc, Pittsburgh, PA USA; 2 JUUL Labs, Inc, Washington, DC USA

**Keywords:** Cigarettes, Electronic nicotine delivery systems, Retail data, Substitution, Trends

## Abstract

Electronic nicotine delivery systems (ENDS) are a potentially lower-risk tobacco product which could help smokers switch completely away from cigarettes. However, the lack of strong evidence to date of a measurable, population-level effect on reducing smoking has generated skepticism about ENDS’ potential benefits. This study examines whether increased US ENDS sales have been associated with reduced cigarette sales. Retail data on weekly per-capita cigarette and ENDS purchases in the USA during 2014–2019 were obtained from a national sample of brick-and-mortar retail outlets. Trends in cigarette sales were modeled before (2014–2016) ENDS had a substantial market share, and, after adjusting for macroeconomic factors, projected into the post-period (2017–2019). Actual cigarette sales were lower than projected sales (by up to 16% across the post-period), indicating a substantial “cigarette shortfall” in the post-period. To explore whether general (i.e., inclusive of potentially many mechanisms) substitution by ENDS can explain the cigarette shortfall, its association with per-capita ENDS volume sales was examined. Higher ENDS sales were significantly associated with a greater cigarette shortfall: for every additional per-capita ENDS unit, cigarette sales were 1.4 packs-per-capita lower than expected (*B* = 1.4, *p* < .0001). Error correction models which account for spurious correlation yielded similar results. These findings support ENDS serving as a substitute for cigarettes (through potentially many mechanisms including cigarette price), causing cigarette consumption to decline. Since ENDS potentially pose lower risk than cigarettes, this general substitution effect suggests that risk-proportionate tobacco regulation could mitigate the tobacco-related health burden.

**Supplementary Information:**

The online version contains supplementary material available at 10.1007/s10603-022-09533-4.

Cigarette smoking remains the leading preventable cause of morbidity and premature mortality, contributing to 480,000 excess deaths in the USA annually (U.S. Department of Health Human Services, 2004, 2014). Since the harmful effects of cigarettes predominantly come from combustion (U.S. Department of Health Human Services, 2014) rather than nicotine (McNeill et al., 2018; Royal College of Physicians of London, 2016), noncombustible nicotine products such as electronic nicotine delivery systems (ENDS) have the potential for harm reduction among smokers (McNeill et al., 2018; National Academies of Sciences Engineering & Medicine, 2018; Royal College of Physicians of London, 2016).

Behavioral studies demonstrate that many smokers use ENDS to completely switch away from smoking. Randomized clinical trials show ENDS to be more effective for smoking cessation than nicotine replacement therapy (NRT) (Hajek et al., 2019; Hartmann-Boyce et al., 2021; Walker et al., 2020), though clinical cessation treatment may have a limited impact at the population level. A potentially greater impact could come from “unplanned switching” among smokers who may not have explicitly intended to quit smoking using ENDS. In a naturalistic study of established smokers who purchased a JUUL starter kit, 51.2% had switched completely away from smoking 12 months later (Goldenson et al., 2021). Established smokers who use ENDS—especially daily—make more quit attempts and are less likely to subsequently smoke (Johnson et al., 2019). Even among those who have not (or not yet) completely switched, dual use of ENDS with smoking is often accompanied by reduced cigarette consumption (Selya et al., 2021) along with reduced exposure to harmful and potentially harmful constituents (Cohen et al., 2021; Goniewicz et al., 2018). At the population level, the decline in smoking prevalence *accelerated* after ENDS became widely available (Foxon & Selya, 2020; Levy et al., 2019). Thus, ENDS appear to offset, reduce, or replace cigarette consumption to some degree (collectively referred to here with the umbrella term “general substitution effect”).

One expression of the substitutability of ENDS for cigarettes is the evidence that ENDS have an *economic* substitution effect specifically, whereby demand for one product increases in response to changes in consumer cost (e.g., higher prices, regulatory barriers) of a different product which consumers view as an alternative product. Quasi-experimental studies of taxation effects show that higher *e-cigarette* prices result in more *cigarette* sales (Cotti et al., 2022; Saffer et al., 2020), especially among younger adults (< 40 years old) (Pesko et al., 2020); this economic substitution effect could be as strong as 2.1 additional packs of cigarettes being purchased for every e-cigarette pod that taxes avert (Cotti et al., 2022). Cigarette consumption also increases following other types of e-cigarette restrictions besides taxes, namely indoor vaping bans (Cooper & Pesko, 2017), restrictions on e-cigarette advertising (Dave et al., 2019a, 2019b; Tuchman, 2019), and minimum legal sale ages (Dave et al., 2019a, 2019b; Friedman, 2015; Pesko et al., 2016) (though see Abouk and Adams (2017)). Behavioral purchasing experiments similarly support an economic substitution effect (Johnson et al., 2017; Snider et al., 2017; Stein et al., 2018).

Though these studies collectively provide strong evidence that ENDS serve as a general substitute for cigarettes (including economic substitution mechanisms), less is known about whether these findings lead to tangible population-level effects. For example, a recent letter from several public health groups to the FDA regarding e-cigarette regulation expressed skepticism about ENDS’ potential benefits to adult smokers, citing the current lack of strong evidence that ENDS have made a population-level impact on reducing smoking in some form (prevalence, per-capita consumption, etc.) (American Academy of Pediatrics et al., 2021). A recent study provided some evidence of such an effect using national survey data, showing that adult smoking prevalence declined faster after ENDS became available (Foxon et al., 2022). However, the existing data on a general substitution effect at the population level are based on behavioral survey data, which is infrequent (e.g., annual) and limited by self-report. Retail data, on the other hand, are an underutilized and valuable source of national tobacco use trends. Retail data in Japan show that cigarette sales declined faster after heated tobacco product use became common (Cummings et al., 2020). However, temporal granularity was low (i.e., annual estimates), allowing only identification of an inflection point in cigarette sales, and the findings in Japan—which has a very different tobacco regulatory environment than the USA—may not generalize. Retail data in the USA have several advantages, such as high temporal granularity (namely, weekly data) and the ability to track national-level cigarette and ENDS purchasing. Retail data have been similarly used in the context of health policy in other settings such as sugar-sweetened beverage taxes (Colchero et al., 2017) and removing cigarettes from pharmacies (Sussman, 2015).

The current study examines the US national-level association between ENDS sales and cigarette sales—with the intention of examining the overall association between the two (referred to here as “general substitution”), rather than analyzing the multiple potential mechanisms that may underlie such an effect—using retail data drawn from a large, national sample of brick-and-mortar retail outlets (“tracked channels”) (Bronnenberg et al., 2008) throughout 2014–2019. Cigarette sales (packs per capita) from 2014 to 2016 (before ENDS had a large market share) were modeled as a function of a time trend and macroeconomic factors (quarterly gross domestic product (GDP) and monthly unemployment), and projected into the post-period (2017–2019). These counterfactual projections (i.e., cigarette sales expected in the absence of ENDS) were used to estimate the discrepancy between projected and actual cigarette sales (“cigarette shortfall”). In order to evaluate the potential role of ENDS sales in explaining the cigarette sales shortfall, its association with ENDS sales (units per capita) was examined. The question of whether ENDS serves as a general substitute for cigarettes has important implications for tobacco policy, specifically how ENDS and cigarettes should be regulated and taxed with respect to each other.

## Methods

### Data and Variables

Data on cigarette and ENDS sales were obtained from Information Resources, Inc. (IRI) (IRI, 2002). IRI collects sales data of products to end-consumers using scanner data from a national sample of brick-and-mortar retail outlets. These data are widely used in research on national consumption trends (Kruger, 2020), including tobacco products (Ali et al., 2020; Mayne et al., 2017). This study uses data from all available retail store channels tracked by IRI (i.e., food, grocery, drug, mass merchandiser, club, dollar, military, and convenience stores); notably, this does not include online sales or specialty vape/tobacco stores. Overall, IRI data capture a large majority of the cigarette market (e.g., approximately 85% of sales reported by the US Alcohol and Tobacco Tax and Trade Bureau (The Alcohol and Tobacco Tax and Trade Bureau)), but likely less of the ENDS market (e.g., approximately half the ENDS sales volume compared to Euromonitor (Euromonitor International 2020)). Nevertheless, IRI data importantly capture the *rise* in ENDS use over the time period of interest (Altria Group, 2020), supporting their use for examining the *presence* if not the exact *magnitude* of a general substitution effect between ENDS and cigarettes (see “Limitations”). IRI data between 1 January 2014 (the earliest data available to the authors), and 31 December 2019, were analyzed.

Annual population estimates were obtained from the US Census, and seasonally adjusted monthly unemployment rates from the US Bureau of Labor Statistics.

### Measures

To account for population growth, cigarette sales data were denominated as the weekly per-capita number of cigarette packs (i.e., IRI total cigarette packs purchased, divided by the annual total US population estimate). Per-capita sales are an established source of national-level retail data for analyzing *trends* of other consumable products (Kerr et al., 2006; Shrapnel & Butcher, 2020).

ENDS sales were defined as per-capita weekly number of “units,” approximately representing one unit ENDS product (e.g., one pod, e-liquid bottle, reusable device, or disposable device), in order to focus on a behaviorally relevant measure of ENDS consumption (but see “Limitations” for the implications of this heterogeneous measure).

Covariates included quarterly GDP per capita and the monthly US unemployment rate. Given our primary goal of evaluating the presence and approximate magnitude of an aggregate-level association between ENDS and cigarettes—which could encompass many different mechanisms acting in concert—our main analysis did not control for specific mechanisms, as they would explain away (part of) the very association we are examining. However, supplementary analyses additionally control for cigarette pack price—one likely mechanism of the overall effect—as a robustness check (Supplementary Analysis 2).

### Analyses

**Fig. 1:**
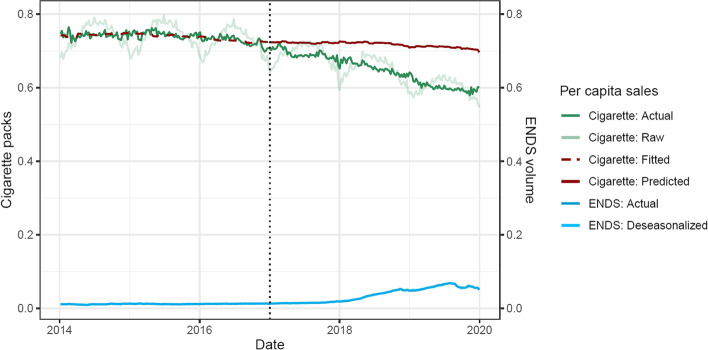
Actual sales of cigarette packs and ENDS units per capita, and projected cigarette sales. Note. Cigarette sales: packs per capita of actual (green) sales, and fitted/predicted sales (red dashed/solid). ENDS sales: units per capita (blue). Solid lines: de-seasonalized trends. Faded lines: raw data. Predicted values are based on fitted values (dashed red line) to a model run on the pre-period data (2014–2017) and projected (solid red line) into the post-period (2017–2019). Dotted vertical line: cutoff between pre-period and post-period

The cigarette and ENDS sales time series were split into two equal periods: a pre-period spanning 2014–2016, when ENDS sales were consistently low; and a post-period spanning 2017–2019, when ENDS market share began to increase (see Fig. 1). Thus, the pre-period covers a time when national ENDS use was low, and the market was comprised of earlier-generation products that were less effective at nicotine delivery (Voos et al., 2019); and the post-period covers the rise of newer-generation, higher-nicotine products. This cutoff is approximate given that ENDS sales show a gradual increase, and as a result, sensitivity analyses vary the cutoff between pre- and post-periods (see below).

Cigarette and ENDS sales data were de-seasonalized (i.e., removing consistent variation over each year, such as higher cigarette sales in summer months and lower sales in winter months (Chandra & Chaloupka, 2003)) prior to analysis, as seasonal variation can introduce spurious correlation. De-seasonalization is a statistical method that can be used to filter out any systematic variations showing a cyclical pattern over the period of 52 weeks (without needing to explicitly measure or specify particular sources of this variation, e.g., climactic condition). This was performed using multiplicative decomposition of cigarette and ENDS sales data. ENDS did not show the same prominent seasonality as cigarette sales (Fig. 1)—possibly due masking by the large increase in overall ENDS sales—but were de-seasonalized nevertheless to avoid the possibility of spurious correlation due to common seasonal factors.

To estimate counterfactual cigarette sales—i.e., what trends would have been in the absence of substantial ENDS uptake—a time series linear model (ordinary least squares (OLS)) was first fit on the pre-period, as a function of de-seasonalized trend and macroeconomic factors (GDP and unemployment), and this model was extrapolated into the post-period (2017–2019) to generate *projected* cigarette sales. The shortfall in cigarette sales was calculated as the difference between actual and projected cigarettes sales. Data from 2020 onward were excluded due to shocks introduced by COVID-19 (e.g., increased unemployment, store closures) which obscure the relationship between smoking and ENDS use.

To evaluate how well ENDS volume sales may explain the cigarette sales shortfall, OLS regressions examined the relationship between the cigarette sales shortfall and per-capita ENDS sales over the post-period (2017–2019). Follow-up analyses were performed to account for the possibility of a spurious association, which can result from both variables having trends over time (see Supplementary Analysis 1). One way of ruling out spurious correlation is to examine the changes between successive time points, and whether the variables show a tendency to converge to an equilibrium relationship following a perturbation in one or both variables. That is, when there is a large “error” in the relationship (resulting in discrepancies between actual and projected values), do the variables then subsequently change in a way that *reduces* that error (hence, the term “error correction”) and return towards their long-term equilibrium relationship? The possibility of such an equilibrium relationship between the cigarette sales shortfall and ENDS sales variables was examined using an error correction model (ECM), which would indicate a non-spurious relationship.

Several robustness checks were performed. First, sensitivity analyses varied the duration of the pre-period by 6 months (i.e., ending at 30 June 2017) and by one year (i.e., ending at 31 December 2017), and correspondingly shortened the post-periods. Supplementary analyses additionally controlled for cigarette pack price (Supplementary Analysis 2), and ran the main analyses within each of four regions in the USA (Supplementary Analysis 3).

## Results

Figure 1 shows the de-seasonalized trends in per-capita cigarette sales and ENDS sales (solid green and blue lines, respectively). Cigarette sales trends decrease over time, especially in the post-period (right of the dotted vertical line), with a slight uptick at the end of 2019. Correspondingly, de-seasonalized ENDS sales in IRI tracked channels were very low until 2017, then began increasing through mid-2019, before partially declining. Raw cigarette sales show substantial seasonality (faded green line), increasing in summer months and decreasing in winter months, with an overall declining trend, while raw ENDS sales show no prominent seasonality (and was nearly identical to the de-seasonalized time series).

The time series linear model fits the pre-period (2014–2016) data well (*r* = 0.66, adjusted *R*^2^ = 0.44, residuals approximately normally distributed, mean absolute percent error (MAPE) = 1.0%; Fig. 1, dashed red line vs. solid green line), indicating that the variation in cigarette sales from 2014 to 2016 is well-explained by a significant trend component (annual decline of 0.02 packs per capita, *p* < 0.0001), and unemployment rate (each percentage point was associated with a decline of 0.02 packs per capita, *p* < 0.0001), but not GDP (*p* = 0.485).

Counterfactual projections of cigarette sales (2017–2019) (Fig. 1, solid red line) show a significant and growing shortfall with actual cigarette sales (Fig. 1, solid red vs. green lines). Actual sales fall substantially below projected sales by up to 0.11 packs per capita (15.8% lower sales) across the post-period, and fit the predicted values poorly (MAPE = 9.1%).

**Fig. 2:**
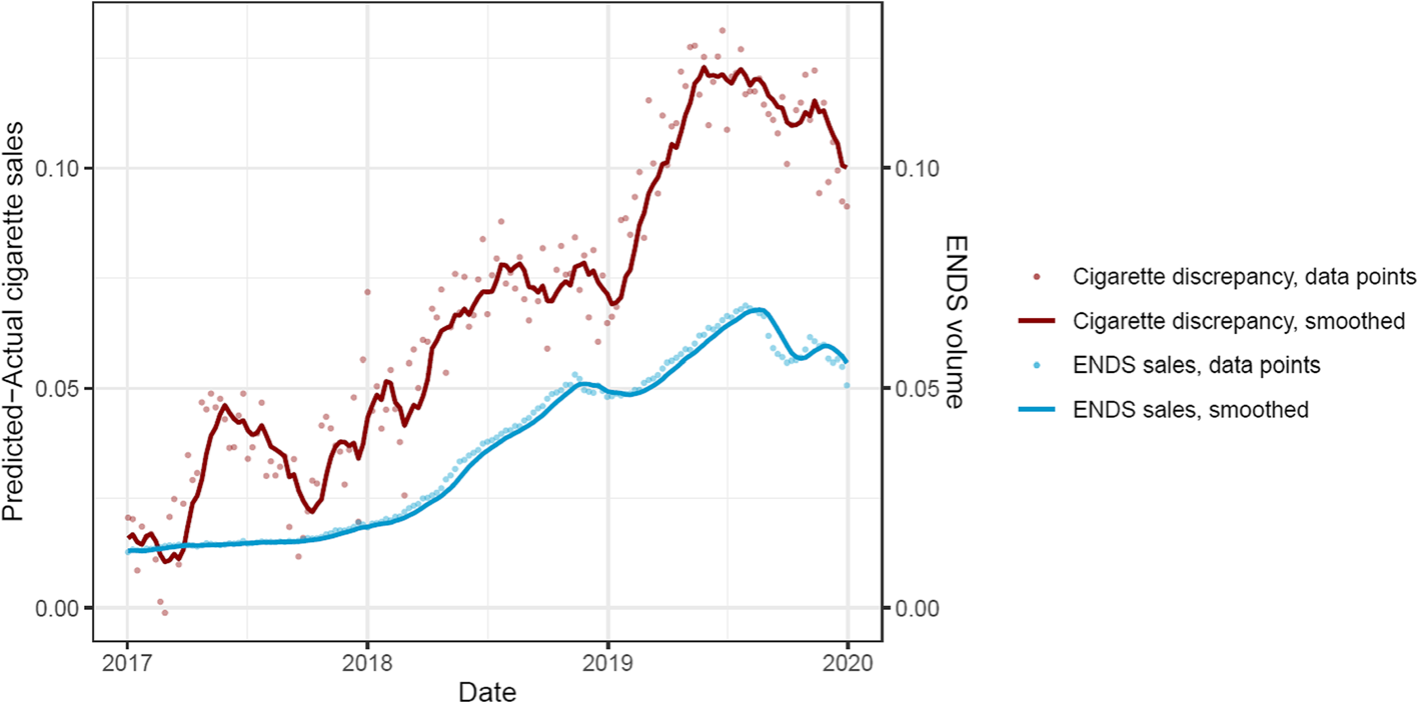
Cigarette discrepancy and ENDS sales per capita. Note. Cigarette discrepancy: discrepancy between actual and projected de-seasonalized per capita cigarette packs sales (red), with more positive values indicating a greater shortfall in cigarette sales. ENDS sales: de-seasonalized, per capita ENDS volume purchases (blue). Solid lines: smoothed data. Faded points: raw data

The growing cigarette sales shortfall suggests the presence of another factor in the post-period that may affect cigarette pack sales. Per-capita ENDS volume was compared with the cigarette sales shortfall (where more positive values mean a greater shortfall) (Fig. 2); for every 1-unit per capita increase in ENDS sales, the shortfall in cigarette sales significantly increased by approximately 1.4 packs per capita (*B* = 1.4, *p* < 0.0001).

Supplementary analyses that tested for potential spurious correlation showed that, despite significant trends in both the cigarette shortfall and ENDS sales variables, their combination was stable over time, indicating a long-run equilibrium relationship. OLS estimates are statistically consistent given these characteristics of the data and the sufficient sample size, suggesting that the main analysis above is robust. Furthermore, an ECM appropriate for analyzing relationships between variables with these characteristics produced a similar estimate: every 1-unit increase in per-capita ENDS sales is associated with a 1.5 packs-per-capita shortfall in cigarette sales (see Supplementary Analysis 1). The ECM further showed that the cigarette shortfall responds to changes in ENDS sales rather than vice versa, consistent with ENDS sales being the driving force. These supplementary analyses reinforce the main findings and suggest an equilibrium relationship indicative of ENDS substituting for cigarettes.

Table 1 presents the main and sensitivity results. Analyses using longer pre-periods also showed that actual cigarette sales were lower than projected, though the magnitude of this shortfall was smaller versus the primary analysis (see “Discussion”). For all analyses, the shortfall in cigarette sales was positively associated with ENDS sales across the post-period. Each additional per-capita ENDS volume was associated with a 0.7–0.9 per-capita decrease in cigarette pack sales (*p* < 0.0001 in all cases) across the sensitivity analyses.

**Table 1:** Results of primary and sensitivity analyses

Analysis	Pre-period end	Maximum discrepancy over post-period		Change in cigarette discrepancy per 1-unit increase in ENDS
		Per capita packs	Percentage	
Primary analysis	Dec 31, 2016	0.11	15.8%	*B* = 1.4 *p* < .0001
Sensitivity analysis 1	June 30, 2017	0.07	10.6%	*B* = 0.9 *p* < .0001
Sensitivity analysis 2	Dec 31, 2017	0.04	6.7%	*B* = 0.6 *p* < .0001

Finally, additional robustness checks were performed as supplementary analyses. First, we additionally controlled for average cigarette pack price—one specific mechanism of the general substitution—which produces similar but slightly smaller effects (Supplementary Analysis 2). We also re-analyzed these associations within each of 4 US Census regions, finding similar results in 3 regions and inconclusive results in the 4th (Supplementary Analysis 3).

## Discussion

US retail data show that cigarette sales became up to 16% lower than expected as ENDS became common (2017–2019), than what would be expected from prior cigarette sales trends (2014–2016). The shortfall in per-capita cigarette sales was significantly associated with per-capita ENDS sales, such that every additional unit of ENDS volume per capita is accompanied by a *reduction* in cigarette sales of 1.4 packs per capita, based on sales in tracked retail channels. Moreover, a supplementary ECM analysis shows that changes in ENDS sales precede those of cigarette sales, consistent with ENDS being the driver of these changes. Thus, ENDS appear to act as a general substitute for cigarettes.

The goal of the current study was to fill a gap in the current literature by assessing whether ENDS uptake has been associated with a measurable, population-level impact on reducing cigarette smoking; thus, the general substitution effect we report intentionally aggregates across multiple specific mechanisms. While examining specific mechanisms is outside the scope of this study, several potential mechanisms could contribute to this overall, national-level substitution. For example, there is robust evidence of economic substitution between ENDS and cigarettes, as a function of cigarette price (Cotti et al., 2022; Pesko et al., 2020). Local and national policy changes that make cigarettes or ENDS less accessible may also be other mechanisms contributing to the overall substitution effect we report. Local policies can include local flavor bans and smokefree air laws, which both show evidence for a type of substitution in that restrictions on ENDS are associated with subsequent increases in cigarette smoking (Cooper & Pesko, 2017; Friedman, 2022; Friedman, 2021). Additionally, many states and localities raised the legal age of tobacco purchase to 21 years ("T21") throughout the date range examined in the study; T21 decreases cigarette smoking (Abouk et al., 2021) and may have an effect on ENDS use (Abouk et al., 2021). Thus, the literature identifies several specific mechanisms that could explain the aggregate-level general substitution effect we report; future research should examine the role of particular mechanisms in driving this relationship.

The unexpected decline in cigarette sales observed in 2018–2019 has also been noted by financial analysts (Herzog & Kanada, 2018), in news articles (Al-Muslim, 2019; Marcos, 2019), and by cigarette companies (Al-Muslim, 2019; Marcos, 2019). For example, Altria reported a 5.5% decline in cigarette volumes in 2019, and attributed 2.0% to displacement by ENDS sales (Altria Group, 2020).

Raw cigarette sales trends showed prominent seasonality, consistent with prior literature (Chandra & Chaloupka, 2003), while raw ENDS sales did not, suggesting that ENDS do not necessarily substitute for cigarettes in all settings. Cigarette sales may show a seasonal pattern due to smokers forgoing cigarettes to avoid going outside during winter months (Chandra & Chaloupka, 2003; Momperousse et al., 2007), as well as declines for New Year’s resolutions (Chandra & Chaloupka, 2003). It is unclear whether these factors influence ENDS sales, but the lack of seasonality in ENDS sales suggests that the determinants of seasonal fluctuations in smoking are separate from those underlying ENDS’ general substitution effect.

The finding that ENDS sales offset cigarette sales was robust across several sensitivity analyses. Supplemental analyses using ECMs yielded a similar ENDS-driven substitution effect, confirming the main findings that ENDS act as general substitutes for cigarettes. Additionally, sensitivity analyses that extended the date separating the pre-period from the post-period also support a general substitution effect of ENDS, though the effects were smaller. This is expected because the pre-period model is fit to more data points in which ENDS sales are already higher—and have already offset cigarette sales to some degree. Thus, these sensitivity models are more accurate in the post-period, resulting in a smaller shortfall.

Similarly, supplemental analyses show robustness of the main findings. A model additionally adjusting for average cigarette pack price (Supplementary Analysis 2) produced very similar results—but slightly reduced, as adjusting for cigarette pack price—one mechanism of substitution—would be expected to diminish the overall aggregate substitution effect. Analyses within each of four US regions (Supplementary Analysis 3) were also generally consistent with the main findings: the Midwest, South, and West showed a clear cigarette shortfall in the post-period that was correlated with ENDS sales (while the Northeast results were inconclusive due to a discrete event in mid-2016 impairing the model’s ability to detect a significant cigarette shortfall).

The current study estimates ENDS’ general substitution effect to be 1.4–1.5 packs per capita for every per-capita ENDS unit; however, this estimate lacks the contribution of other sales channels not tracked in the current data (i.e., online sales and specialty vape/tobacco store sales). Furthermore, the units of ENDS products are heterogeneous, measuring devices and nicotine pods of different sizes all as equal units. Accordingly, this estimate should be interpreted as an imprecise one.

The finding of a significant general substitution effect between ENDS and cigarettes is consistent with previous quasi-experimental work showing economic substitution—one likely mechanism contributing to the overall substitution effect we report—in response to tobacco taxes (Cotti et al., 2022; Pesko et al., 2020; Saffer et al., 2020) and other types of restrictions on e-cigarette purchasing (Dave et al., 2019a, 2019b; Friedman, 2015; Pesko et al., 2016). On the other hand, Allcott & Rafkin, (2021) showed *no* economic substitution in response to ENDS taxes; however, their data (Nielsen retail data) only cover 2.5% of national e-cigarette sales, and as such may not be representative. Furthermore, they examine this one very specific mechanism of substitution, while the current study aggregates across potentially many mechanisms. The economic substitution reported in this literature is likely but one contribution to the aggregate-level substitution effect reported here.

The current findings are also consistent with behavioral literature showing that smokers have high rates of switching away from cigarettes after adopting ENDS (Goldenson et al., 2021). A similar study showed a substitution effect between NRT and cigarettes using national retail data (Chandra et al., 2011). Even among ENDS users who do not completely switch away from smoking, the majority significantly reduce their cigarette consumption (Selya et al., 2021). Also consistent with the current findings, similar work examining actual versus projected smoking *prevalence* shows that actual smoking declined faster after ENDS than would be expected from pre-existing trends (Foxon & Selya, 2020; Levy et al., 2019). Thus, ENDS appear to be diverting people away from smoking at the population level, thereby contributing to record low smoking rates (Foxon & Selya, 2020; Foxon et al., 2022; Levy et al., 2019; Selya & Foxon, 2021). The cigarette sales data presented here would reflect both reductions in the *number* of smokers and the *amount* smoked among remaining smokers.

These findings have important implications for public health and policy. Considering ENDS likely offer adult smokers lower risk compared to cigarettes (McNeill et al., 2018; National Academies of Sciences Engineering & Medicine, 2018), population health stands to benefit from a shift away from cigarettes towards ENDS; e.g., one study estimated that this could save 113.2 million life-years by 2,100 (Warner & Mendez, 2020). On the other hand, youth use is a concern: although youth ENDS use has declined dramatically since 2019 (Park-Lee et al., 2021), the concern over youth use continues to motivate heavier restrictions on ENDS that impact adult smokers as well. Policymakers are thus faced with a difficult policy challenge of balancing the risks and benefits of ENDS (Balfour et al., 2021). However, overly restrictive ENDS regulation (relative to cigarette regulation) has detrimental unintended consequences, namely increased smoking rates for youth (Friedman, 2015) and adults alike (Pesko et al., 2020) underscoring the general substitution effect we observe here. Moreover, modeling under a wide range of assumptions suggests that the availability of ENDS *saves* life-years overall, even under unrealistically pessimistic assumptions (Warner & Mendez, 2019). The potential net public health gain from ENDS substituting for cigarettes is consistent with the tobacco harm reduction principles enunciated by the US Food and Drug Administration (FDA) (US Food and Drug Administration). Together, this suggests that the tobacco-related health burden could be mitigated through risk-proportionate tobacco policy (Balfour et al., 2021), which would disincentivize cigarettes (e.g., through higher taxes) more relative to ENDS to encourage switching among adult smokers, while implementing targeted measures to deter youth use (e.g., strictly enforced age controls and marketing restrictions).

### Limitations

The data used in the present analyses did not include online sales or specialty vape/tobacco shops, and thus not all ENDS sales are represented here, particularly for open tank products which are primarily sold in specialty vape stores (Braak et al., 2019). Additionally, some of the apparent decline in cigarette sales could reflect a shift away from purchasing in brick-and-mortar retail outlets, towards untracked channels. Given that IRI capture ~ 85% of cigarette sales and ~ 50% of ENDS sales, the full substitution effect may be smaller than our estimate (as approximately the same cigarette decline would be “diluted” by more ENDS sales). Relatedly, it is possible that smokers may be switching to other products not accounted for here. It is also possible that total ENDS sales (tracked and untracked) could be flat or rising less slowly than IRI-tracked ENDS sales indicate; however, IRI trends are consistent with behavioral survey trends, which also show ENDS use rising throughout 2017–2019 (Dai & Leventhal, 2019), suggesting that IRI data approximately capture true trends. Nevertheless, although the exact magnitude of ENDS’ full substitution effect may be smaller than reported here, that ENDS are a substitute for cigarettes has robust support in this and previous studies (Johnson et al., 2017; Snider et al., 2017; Stein et al., 2018).

The measure of ENDS sales in terms of ENDS *units* also has limitations, in that this does not capture the underlying heterogeneity of ENDS products (i.e., disposables, pods, refillable devices). This introduces imprecision into the effect estimates. Nevertheless, ENDS units are the best behaviorally relevant measure of ENDS consumption available in these data.

The data presented are per-capita estimates of ENDS and cigarette sales; as such, these results cannot distinguish between complete switching away from cigarettes and reducing (but not eliminating) cigarette consumption via ENDS. This distinction has public health implications: while substantial smoking reduction after adopting ENDS is shown to reduce exposure to harmful and potentially harmful constituents (Cohen et al., 2021; Goniewicz et al., 2018), greater harm reduction would be expected from switching completely away from cigarettes (Cohen et al., 2021; Goniewicz et al., 2018; Jay et al., 2020; McEwan et al., 2021). The data also do not distinguish sales made to adult smokers from those made to non-smokers, including non-smoking youth, which may impact the public health implications.

Our main analyses do not establish a causal relationship between ENDS sales and the shortfall in cigarette sales; however, it seems unlikely that there are other strong determinants of cigarette sales from 2017 that could explain this shortfall. As discussed above, changing tobacco regulations could impact both cigarette and ENDS use; however, these effects may be among several other potential mechanisms underlying the overall, aggregate-level substitution effect we report. Other unrelated drivers (e.g., other measures of economic environment) may explain part of the current results, but are unlikely to fully account for the association between ENDS sales and the cigarette shortfall. Additionally, the ECM in the Supplement suggests that changes in ENDS sales precede the shortfall in cigarette sales, rather than vice versa, supporting the role of ENDS in explaining the cigarette sales shortfall.

Similarly, current analyses do not account for all possible determinants of cigarette and ENDS purchasing behavior, such as state and local variation in tobacco taxes and policies or additional macroeconomic factors such as inflation. However, this was intentional, consistent with our primary goal of examining a potential aggregate, national-level association between ENDS and cigarette sales, which may be due to multiple potential mechanisms: adjusting for specific potential mechanisms would explain away the very association we are examining. Additional research is needed to identify the mechanisms of the overall substitution effect. Another disruption may have been due to the “e-cigarette or vaping associated lung injury” (EVALI) outbreak in late 2019, which was initially attributed to (nicotine) e-cigarettes but was later recognized to be caused by vitamin E acetate, an additive to illicit THC vapes (Office on Smoking and Health & National Center for Chronic Disease Prevention and Health Promotion). Despite the cause not being related to nicotine e-cigarettes, people’s risk perceptions of e-cigarettes significantly worsened following the initial messaging of EVALI and only partially recovered after the true cause was announced (Dave et al., 2020). These omitted factors may explain some of the reported general substitution effect. However, given the robustness of the reported substitution effect, these additional factors are unlikely to change the main finding of a significant general substitution effect between ENDS and cigarettes. Having established the existence of a national-level, aggregate substitution effect, future research is needed to rigorously examine the underlying mechanisms.

### Strengths

This study is novel in its use of national retail data with a high degree of temporal resolution. The findings were robust across several different sensitivity analyses, including rigorous ECMs that account for the possibility of spurious correlation. Sensitivity analyses were conducted to vary the length of the pre- and post-periods over which to model and project cigarette sales, and several robustness checks were conducted (Supplementary Analyses 2 and 3).

## Conclusions

Increased ENDS sales were significantly associated with otherwise unexpected declines in cigarette sales, showing a possible population-level impact of ENDS on reducing smoking. This provides evidence that ENDS are acting as a general substitute for cigarettes, possibly contributing to the decline in national cigarette consumption. Considering the potential that ENDS provide adult smokers reduced risk compared to cigarettes, shifting adult smokers who would not otherwise quit from cigarettes to ENDS is consistent with reducing tobacco-related harm in the US population. In support of this goal, risk-proportionate tobacco regulations are needed to differentially disincentivize more harmful combustible products.

## Supplementary Information

Below is the link to the electronic supplementary material.

Supplementary file1 (DOCX 403 KB)
