# Supplementary material for: Sales of Electronic Nicotine Delivery Systems (ENDS) and Cigarette Sales in the USA: A Trend Break Analysis
Source: J Consum Policy (Dordr). 2023 Jan 16;46(1):79–93. doi: 10.1007/s10603-022-09533-4 (PMC9841499; doi:10.1007/s10603-022-09533-4)
Supplement: Supplementary file 1 — Supplementary file1 (DOCX 403 KB) [file 10603_2022_9533_MOESM1_ESM.docx]

**SUPPLEMENT**

**Supplemental Analysis 1: Error Correction Model**

**Methods & Results**

It is possible for two time series variables that are in fact unrelated to be correlated, leading to the incorrect inference that there is a relationship between them. This is called the spurious regression problem (Granger & Newbold, 1974), and it often occurs when the two series in question show a high degree of persistence over time. A standard practice in econometric analysis is to study the properties of each time series individually and in combination before attempting to estimate and interpret any relationships between them.

In the first step of our analysis, therefore, we tested each time series (the cigarette sales *shortfall*, i.e. projected minus actual sales; and ENDS sales) for the presence of a unit root, or a variation over time in the time series’ statistical properties. When a time series shows a unit root, it can show spurious intercorrelations with other variables. Both time series displayed evidence of unit root processes. When the individual time series have a unit root, valid relationships can still be discerned if their sum (or other linear combination) does *not* display a unit root (i.e. is stable or stationary over time). In this case, the two series are said to be co-integrated, meaning that the variables tend to converge to an equilibrium relationship. In other words, co-integration means that if there is a large ‘error’ or perturbation in the relationship, that one or both variables subsequently change to reduce that error and thus return to their long-run equilibrium relationship. When individual time series show a unit root and are co-integrated, OLS analyses of these time series (as performed in the main manuscript) are valid without any additional steps such as first-differencing (Engle & Granger; Granger, 1981; Hamilton, 1994); this is standard practice in econometrics. A test of cointegration indicated that the cigarette sales shortfall and ENDS sales variables were indeed cointegrated. Therefore, we present the OLS results, which are consistent in the statistical sense, in the main body of the paper.

In this supplement, we present results from a richer model that can be applied to co-integrated series, the error correction model (ECM), which estimates separately the short- and long-term relationships between the two variables in two steps.

In the first step of the ECM, our estimates indicated a cointegrating (i.e., the equation describing the long-run equilibrium) relationship of the form

$Cig Discrepancy-1.54*ENDS Sales=0$.

This indicates that a unit increase in ENDS sales is associated with 1.54 unit increase in the shortfall between projected and actual cigarette sales. This relationship has the same sign and is similar in magnitude to the OLS coefficient of 1.4 in the main manuscript.

Next, we estimated the error correction equation of the ECM relating the cigarette shortfall with ENDS sales. This analysis returns separate coefficients for the error correction erm, a trend term, and the lags of the variables included in the model. Table S1 shows the estimates from the error correction equation of the ECM. In the following discussion, we focus on those parameter estimates that are statistically significant and indicative of a relationship between the two variables.

**Table S1. Results of error correction model, presented as coefficient (SE). Bold: p<.05.**

| Dependent Variable | Error Correction Term | Trend | Lagged ENDS Sales | Lagged Cigarette Shortfall |
| --- | --- | --- | --- | --- |
| Cig Shortfall | **-0.221**  **(0.062)** | 3.2 x 10^-6^ (6.2 x 10^-6^) | **1.395 (0.558)** | **-0.335**  **(0.075)** |
| ENDS Sales | 0.013  (0.009) | 1.6 x 10^-6^ (8.9 x 10^-7^) | **0.274 (0.079)** | 0.008  (0.011) |

The estimates in Table S1 provide a rich picture of the relationship between ENDS sales and the cigarette shortfall time series. First, the error correction term for cigarette shortfall is statistically significant and between 0 and -1 in value, indicating that the shortfall in cigarette sales has a tendency to revert to the long-run equilibrium relationship with ENDS sales, with the negative polarity indicating that the relationship between the variables *counteracts* any disequilibrium in the system, and the magnitude of the coefficient indicating the speed at which this correction occurs. The error correction term for ENDS sales, on the other hand, is small and not statistically significant, indicating that it does not respond to the forces that drive the equilibrium. Taken together, these estimates suggest that the driving variable in the relationship is ENDS sales, and that the shortfall in cigarette sales responds to changes in ENDS sales rather than the other way round. The other interesting contrast between the two models is that changes in lagged ENDS sales are positively associated with changes in the cigarette sales shortfall after controlling for the tendency of the variables return to their equilibrium (as captured by the error correction component of the model), while changes in the lagged cigarette shortfall is not associated with changes in ENDS sales. This provides additional evidence supporting the explanation that changes in ENDS sales may be driving changes in the cigarette shortfall, but not vice versa.

**Summary & Conclusions**

Supplemental analyses which account for the possibility of spurious correlation between the cigarette shortfall variable and ENDS sales show that both time series individually are non-stationary, but are cointegrated such that their combination is at equilibrium, indicative of a long-run, non-spurious relationship. Given these characteristics, the OLS estimates presented in the main manuscript is valid and the estimates are statistically consistent. Further, the estimates from the error correction model showing the relationship between ENDS sales and the cigarette shortfall are statistically significant, have the same (i.e., positive) sign, and similar magnitudes to the magnitude of the effect in the OLS model. These supplemental analyses reinforce the main findings of a significant substitution effect between ENDS and cigarettes, with added indications that ENDS Sales seem to be driving the shortfall between predicted and actual cigarette sales.

**Supplemental Analysis 2: Controlling for Cigarette Pack Price**

**Methods & Results**

Although the goal of this paper is to examine the *aggregate*-level substitution effect between ENDS and cigarettes – of which price is likely one of many contributing factors – adjusting for cigarette pack price may be informative. Higher cigarette pack price can deter cigarette use, and thereby increase ENDS use (Cotti et al., 2022; Pesko et al., 2020), explaining one potential mechanism for substitution. Controlling for cigarette pack price can inform on whether cigarette pack price explains some of the aggregate substitution effect observed in the main analysis, and the remaining portion from other substitution mechanisms.

Average cigarette pack price was calculated as the total cigarette revenue at the national level, divided by the total number of cigarette packs sold for that week across the nation; this was then included as an additional covariate (along with unemployment and per-capita GDP, as in the main analysis) in the time series regression model which forecasts cigarette sales. As in the main analysis, the shortfall in cigarette sales was calculated as the difference between the forecasted and actual cigarette sales. OLS regression was then used to examine the association between ENDS sales and the cigarette shortfall: this is appropriate given the time series’ characteristics (i.e. each has a unit root and together are cointegrated; see Supplementary Analysis 1), as in the main analysis.

This time series linear model fit the pre-period (2014-16) data well, and similarly to the main analysis (r=0.67, adjusted R^2^= 0.44, residuals approximately normally distributed, MAPE=1.0%). Unemployment rate was significantly associated with cigarette sales (each percentage point was associated with a decline of 0.02 packs per capita, *p*<.0001), but the other variables (overall trend, per capita GDP, and cigarette pack price) were not significant in this model.

Nevertheless, because cigarette pack price showed a trend in explaining cigarette sales (with each $1 increase in price being associated with 0.07 fewer packs per capita, *p*=.0605), the remaining analysis was carried out, using projections that now include cigarette price, and regressing the resulting cigarette shortfall onto ENDS sales.

Like the main model, there was a significant and growing shortfall in cigarette sales, by up to 0.09 packs (13.7% lower sales) across the post-period, and the model’s predictions fit the actual data poorly over the post-period (MAPE=7.9%). OLS regression showed that ENDS sales were significantly associated with the shortfall in cigarette sales, such that every per-capita increase in ENDS sales was associated with a 1.3 packs-per-capita shortfall in cigarette sales (B=1.3, *p*<.0001).

**Summary & Conclusions**

This supplemental analysis that additionally controls for cigarette pack price shows similar results as the main analysis, with slightly smaller cigarette discrepancies (13.6% shortfall in cigarette sales vs. 15.8% in the main analysis) and a slightly weaker remaining association between ENDS sales and the cigarette sales shortfall (B=1.3 packs-per-capita shortfall for each per-capita unit of ENDS sales, vs. 1.4 in the main analysis).

This is as expected since cigarette pack price is one of several possible mechanisms of the aggregate-level substitution effect between ENDS and cigarettes. That is, higher cigarette prices deter cigarette purchasing and encourage ENDS purchasing through economic substitution (Cotti et al., 2022; Pesko et al., 2020). Thus, accounting for this aspect of overall aggregate-level substitution slightly diminishes the remaining substitution effect, while confirming that cigarette pack price does marginally explain part of the overall substitution effect between cigarettes and ENDS. Including this covariate does not change the key conclusions of the main analysis: in the aggregate, higher ENDS sales is associated with increases the sales discrepancy for cigarettes.

**Supplemental Analysis 3: By US Census Region**

**Methods & Results**

In order to evaluate the consistency of the findings using national US data, here we repeat the main analyses within each of the 4 US census regions. IRI covers 44/50 US states (see Table S2), with the non-covered states being Alaska, Delaware, Hawaii, Idaho, Montana, and New Jersey. Most of these states (37/44) have data available throughout the full date range in this study (2014-19); however, 7 states (Connecticut, Kansas, Minnesota, Nebraska, Mississippi, New Mexico, and Wyoming) were not tracked until several years into this study’s date range (2016-19). These 7 states were excluded from this supplementary analysis, since they had limited or no data in the pre-period from which to generate projections into the post-period, and since new states entering the data pool could produce artificial changes in sales. Table S2 shows the final list of states within each US census region that were covered by IRI for the full date range in this study. State-level data are available only from the convenience store channel, in contrast to the main analysis of national-level data which also included other retail channels (drug, food, and mass merchandizer stores); this is not expected to impact overall trends.

**Table S2: US Census Regions and Corresponding States and Dates of Data Availability**

| **US Census Region** | **States Covered by IRI throughout full date range*** |
| --- | --- |
| Midwest | Illinois |
|  | Indiana |
|  | Iowa |
|  | Michigan |
|  | Missouri |
|  | North Dakota |
|  | Ohio |
|  | South Dakota |
|  | Wisconsin |
| Northeast | Maine |
|  | New Hampshire |
|  | Massachusetts |
|  | New York |
|  | Pennsylvania |
|  | Rhode Island |
|  | Vermont |
| South | Alabama |
|  | Arkansas |
|  | Florida |
|  | Georgia |
|  | Kentucky |
|  | Louisiana |
|  | Maryland |
|  | North Carolina |
|  | Oklahoma |
|  | South Carolina |
|  | Texas |
|  | Tennessee |
|  | Virginia |
|  | West Virginia |
| West | Arizona |
|  | California |
|  | Colorado |
|  | Nevada |
|  | Oregon |
|  | Utah |
|  | Washington |

* Full date range for this study is Jan 1, 2014 – Dec 31, 2019.

As in the main model, time series linear regressions were performed within each of the four US census regions, and quarterly per-capita GDP and monthly unemployment rate were adjusted for, here using regional versions of these variables. Specifically, regional per-capita GDP was calculated as a weighted average (by state population) across the states comprising each region, and regional unemployment rate was calculated as a weighted average (by state population) of state unemployment rates in each region.

Modeling of cigarette sales in the pre-period (Table S4) generally showed findings similar to those in the main analysis. The regional models for the Northeast and South fit well according to adjusted R^2^ values (.65 and .72), but less so for the Midwest and West (R^2^=0.28 and 0.33, respectively); however, MAPE values in the pre-period was very low and similar across regions. All regional models showed a significant declining trend in cigarette sales (by 0.02 to 0.05 packs per capita annually) and a significant effect of unemployment rate (with each percentage point increase in unemployment being associated with a decline of 0.01 to 0.08 packs per capita). Per-capita GDP associations were less consistent across models (non-significant in the South, negatively associated with cigarette sales in the Midwest, and positively associated in the Northeast and West).

**Table S4: Fit statistics from time series linear regression of per-capita cigarette sales in pre-period (2014-16) in each US Census region.**

| US Census Region | r | Adjusted R^2^ | MAPE for pre-period | Coefficients  B (SE), *p* | | |
| --- | --- | --- | --- | --- | --- | --- |
|  |  |  |  | Annual Trend | Unemployment Rate | Per Capita GDP (per $10,000) |
| Midwest | .53 | .28 | 1.2% | -0.02, (0.003), *p<*.0001 | 0.03 (0.004), *p*<.0001 | -0.12 (0.04), *p*=0.0045 |
| Northeast | .81 | .65 | 1.6% | -0.04, (0.003), *p*=.0001 | 0.03 (0.005), *p*<.0001 | 0.10 (0.03), *p*=0.0004 |
| South | .72 | .53 | 1.4% | -0.05 (0.004), *p*<.0001 | 0.08 (0.008), *p*<.0001 | -0.008 (0.03), *p*=.771 |
| West | .58 | .33 | 1.3% | -0.02 (0.003), *p*<.0001 | 0.008 (0.003), *p*=.0093 | 0.04 (0.01), *p*=.0026 |

Projecting these models into the post-period shows a significant discrepancy between actual and projected cigarette sales (Figure S1) in all regions except the Northeast (presented separately below). As in the main model, the fit between projected and actual data in the Midwest, South, and West was poor in the post-period (MAPE: 6.9% - 9.0% across regions, Table S5), substantially worse than in the pre-period (MAPE: 1.2%-1.4%, Table S4) (see below for Northeast). Cigarette sales reached a maximum shortfall across the post period of between 0.04-0.08 packs per capita in the Midwest, South, and West, corresponding to proportional shortfalls of 10.2%-15.6% (Table S5). The cigarette shortfall appears to track with ENDS sales in these regions (Figure S2).

**Table S5: Projections of cigarette shortfall into post-period (2017-19) for each US Census region.**

| US Census Region | MAPE for post-period | Max cigarette shortfall, packs per capita (%) | Mean cigarette shortfall, packs per capita | OLS of cigarette shortfall on ENDS sales,  B/Elasticity (SE), *p* |
| --- | --- | --- | --- | --- |
| Midwest | 6.9% | 0.07 (10.3%) | 0.02 | B=0.39 (0.05), *p*<.0001 |
| Northeast | 2.1% | 0.02 (5.9%) | 0.002 | B= -0.01 (0.03), *p*=.846 |
| South | 7.0% | 0.08 (11.5%) | 0.02 | Elast.† = 0.71 (0.03), *p*<.0001 |
| West | 9.0% | 0.04 (15.6%) | 0.01 | Elast.† = 0.74 (0.03), *p*<.0001 |

† In these regions, the cigarette shortfall and ENDS were not cointegrated; therefore, OLS was run on first-differenced variables. Additionally, the OLS was run on the natural logarithm of each first-differenced variable, since the linear specification was inappropriate (see text); thus, the results are reported in terms of elasticity (not regression coefficient B as in other regions).

OLS regressions were repeated within each region to examine the association between ENDS sales and the cigarette shortfall (see Table S5), after checking the cointegration of the two time series (i.e. proceeding as in the main analysis if they were cointegrated (see Supplementary Analysis 1), but first-differencing if they were not). Each one-unit increase in per-capita ENDS sales was associated with a cigarette shortfall of 0.39 in the Midwest (in which the cigarette decline and ENDS sales were cointegrated). In the South and West (in which the time series were not cointegrated), the cigarette shortfall and ENDS sales were both first-differenced. OLS was conducted with the natural logarithm of both first-differenced variables, as the linear specification (i.e. OLS with both first-differenced variables in their linear form) was inappropriate (i.e., residuals deviated strongly from normality, there were many outliers, and adjusted R^2^ was very low at <.04 in both regions.) while the log-log specification was appropriate (normally-distributed residuals, adjusted R^2^>0.92 in both regions). In the South and West, ENDS sales were significantly associated with greater cigarette shortfalls: for every 10% increase in per-capita ENDS unit sales, the cigarette shortfall increases by 71% and 74%, respectively (note the different interpretation than the Midwest and national-level models; the South and West results are interpreted as elasticity due to the log-log specification). Thus, the Midwest, South, and West regions support the findings of the main national-level analysis in showing a significant association between higher ENDS sales and a greater cigarette shortfall.


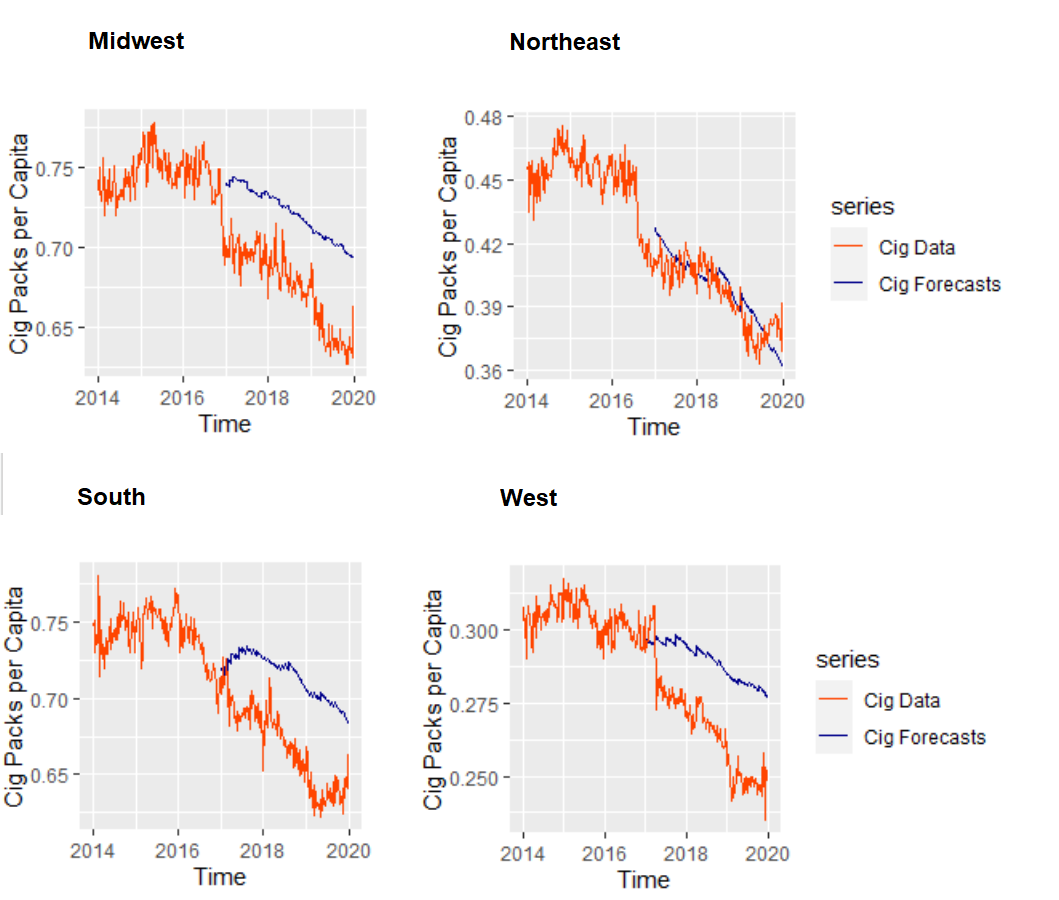


**Figure S1: Actual vs Projected (from pre-period, 2014-16) Per Capita Cigarette Sales in each US Census Region.**


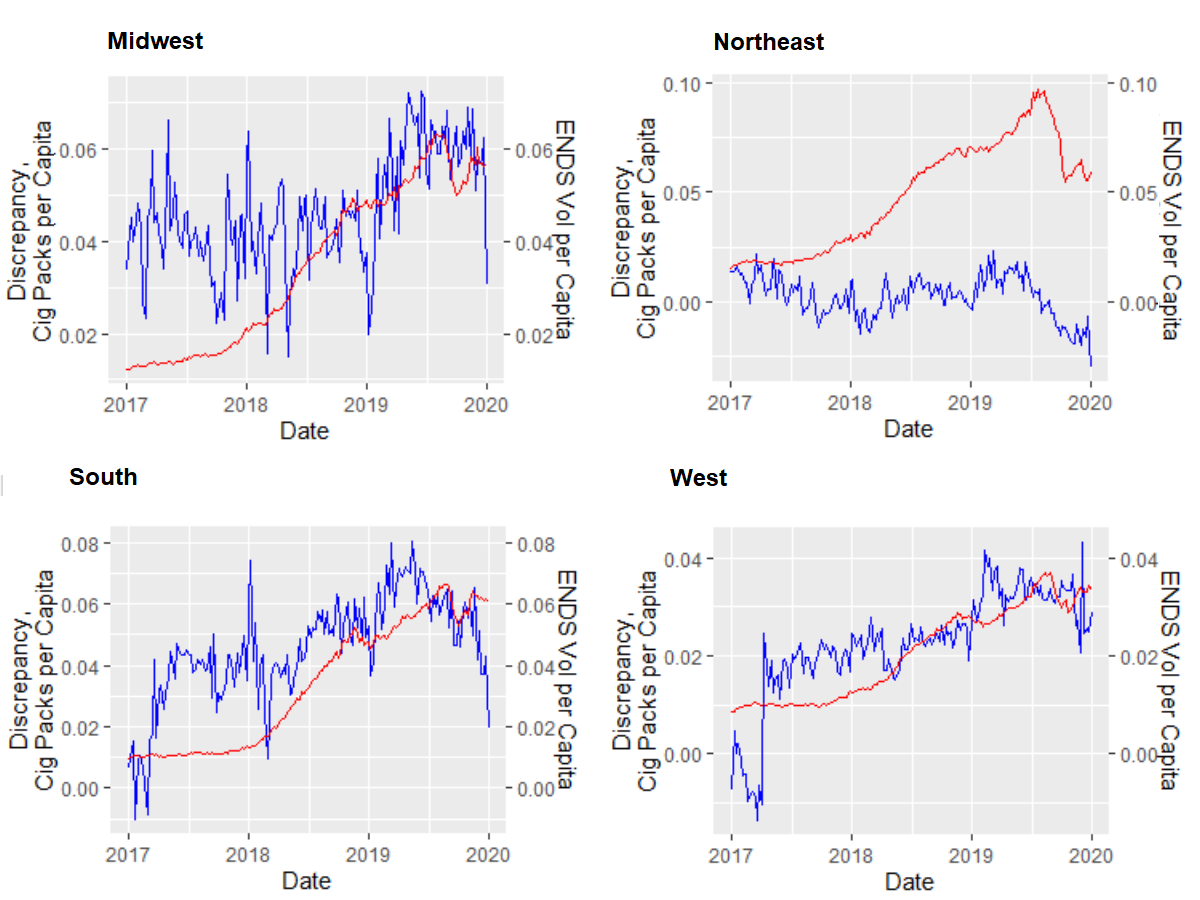


**Figure S2: Cigarette Sales Shortfall and ENDS sales, post-period (2017-19), in each US Census region**

In the Northeast, results differed from the other regions in that the post-period projections fit actual cigarette sales much more accurately (MAPE: 2.1%, versus 1.6% in the pre-period). The shortfall in cigarette sales was approximately zero on average across the post-period, with small and symmetrically distributed deviations in either direction (by less than 0.03 per-capita cigarette packs, or 0.6 individual per-capita cigarettes). A sharp and sudden drop in cigarette sales around mid-2016 is evident in the Northeast cigarette trends (Figure S1). Multiplicative decomposition (not shown) confirms that the “noise” component of the cigarette sales time series is largest immediately before and after this discrete event. This disruption results in *lower* projected cigarette sales compared to a model that excludes the disruption (i.e. by shortening the pre-period; not shown). The (essentially zero) cigarette shortfall is not significantly correlated with ENDS sales. Thus, the impact of the mid-2016 discrete event in the Northeast distorts cigarette sales projections, impairing the ability of the model to detect a significant cigarette shortfall.

**Summary & Conclusions**

All four US Census regions showed declining cigarette sales across the period from 2014-19. For three of these regions (with the exception of the Northeast), higher ENDS sales were significantly associated with a greater shortfall in cigarette sales throughout the post-period, supporting the main findings at the national level.

The Northeast results show a discrete event in mid-2016 that disrupted cigarette sales, impairing the model’s ability to detect a cigarette shortfall. Thus, the Northeast does not unequivocally show the same substitution effect observed in the other US census regions and at the national level; however, such an effect cannot be ruled out – it merely cannot be detected due to the mid-2016 disruption. It is possible that this discrete event may stem from changes in cigarette prices, tobacco policies, etc.; however, since these are potential causal mechanisms of the aggregate-level substitution effect we are examining, a more fine-grained analysis is outside the scope of this study. Instead, understanding specific mechanisms of substitution is a valuable topic for future research with more robust state-level data and more complex methods.

The results in the Northeast also demonstrate sensitivity of the model to the choice of pre- and post-period time windows (i.e., a sudden drop in the pre-period will lower projections into the post-period). The sensitivity analyses that extend the post-period (main manuscript) show that earlier cutoffs produce larger cigarette shortfalls, even in the absence of sudden drops in cigarette sales (as is the case at the national level). Thus, later cutoffs are more conservative (i.e. insensitive), underestimating some of the cigarette shortfall – especially when the pre-period includes a sudden drop.

In regions for which ENDS sales was significantly associated with a cigarette shortfall, the magnitude of the cigarette shortfall in was slightly smaller in the regional analyses compared to the main analysis (0.4-0.8 vs 0.11, respectively). This is expected given the limitations of the state data: namely, that the state data only contain sales from the convenience store channel (versus all available channels at the national level), and thus capture fewer total (and per capita) sales.

In summary, 3 of 4 supplementary regional analyses show the same pattern of results as the main analysis, with the fourth region (the Northeast) being inconclusive due to a discrete event that impaired the model’s ability to detect a cigarette shortfall. Together, these supplementary analyses generally support the main findings that there was a shortfall in cigarette sales in 2017-19, and that ENDS sales were significantly associated with this shortfall, consistent with the explanation that ENDS may drive reduced cigarette consumption in the US.

**References**

Cotti, C. D., Courtemanche, C. J., Maclean, J. C., Nesson, E. T., Pesko, M. F., & Tefft, N. (2022). *The effects of e-cigarette taxes on e-cigarette prices and tobacco product sales: evidence from Retail Panel Data*. <https://www.nber.org/system/files/working_papers/w26724/w26724.pdf> (accessed 29 November, 2022).

Engle, R. F., & Granger, C. W. J. (1987). Co-Integration and Error Correction: Representation, Estimation, and Testing. *Econometrica*, *55*(2), 251-276.

Granger, C. W., & Newbold, P. (1974). Spurious regressions in econometrics. *Journal of econometrics*, *2*(2), 111-120.

Granger, C. W. J. (1981). Some properties of time series data and their use in econometric model specification. *Journal of Econometrics*, *16*(1), 121-130.

Hamilton, J. D. (1994). *Time series analysis*. Princeton University Press. Table of contents <http://www.loc.gov/catdir/toc/prin031/93004958.html> (accessed 29 November, 2022).

Publisher description <http://www.loc.gov/catdir/description/prin021/93004958.html> (accessed 29 November, 2022).

Pesko, M. F., Courtemanche, C. J., & Maclean, J. C. (2020). The effects of traditional cigarette and e-cigarette tax rates on adult tobacco product use. *Journal of risk and uncertainty*, *60*(3), 229-258.
